# Identification and Biochemical Characterization of Halisulfate 3 and Suvanine as Novel Inhibitors of Hepatitis C Virus NS3 Helicase from a Marine Sponge

**DOI:** 10.3390/md12010462

**Published:** 2014-01-21

**Authors:** Atsushi Furuta, Kazi Abdus Salam, Idam Hermawan, Nobuyoshi Akimitsu, Junichi Tanaka, Hidenori Tani, Atsuya Yamashita, Kohji Moriishi, Masamichi Nakakoshi, Masayoshi Tsubuki, Poh Wee Peng, Youichi Suzuki, Naoki Yamamoto, Yuji Sekiguchi, Satoshi Tsuneda, Naohiro Noda

**Affiliations:** 1 Department of Life Science and Medical Bioscience, Waseda University, 2-2 Wakamatsu-cho, Shinjuku-ku, Tokyo 162-8480, Japan; E-Mail: atsushi.5961@ruri.waseda.jp; 2 Biomedical Research Institute, National Institute of Advanced Industrial Science and Technology (AIST), 1-1-1 Higashi, Tsukuba, Ibaraki 305-8566, Japan; E-Mail: y.sekiguchi@aist.go.jp; 3 Radioisotope Center, The University of Tokyo, 2-11-16 Yayoi, Bunkyo-ku, Tokyo 113-0032, Japan; E-Mails: salam_bio26@yahoo.com (K.A.S.); akimitsu@ric.u-tokyo.ac.jp (N.A.); 4 Department of Chemistry, Biology and Marine Science, University of the Ryukyus, Nishihara, Okinawa 903-0213, Japan; E-Mails: damz_98@yahoo.com (I.H.); jtanaka@sci.u-ryukyu.ac.jp (J.T.); 5 Research Institute for Environmental Management Technology, National Institute of Advanced Industrial Science and Technology (AIST), 16-1 Onogawa, Tsukuba, Ibaraki 305-8569, Japan; E-Mail: h.tani@aist.go.jp; 6 Department of Microbiology, Division of Medicine, Graduate School of Medicine and Engineering, University of Yamanashi, 1110 Shimokato, Chuo-shi, Yamanashi 409-3898, Japan; E-Mails: atsuyay@yamanashi.ac.jp (A.Y.); kmoriishi@yamanashi.ac.jp (K.M.); 7 Institute of Medical Chemistry, Hoshi University, 2-4-41 Ebara, Shinagawa-ku, Tokyo 142-8501, Japan; E-Mails: mnakako@hoshi.ac.jp (M.N.); tsubuki@hoshi.ac.jp (M.T.); 8 Department of Microbiology, Yong Loo Lin School of Medicine, National University of Singapore, Center for Translational Medicine, 14 Medical Drive, #15-02, Level 15, Singapore 117599, Singapore; E-Mails: micpwp@nus.edu.sg (P.W.P.); micys@nus.edu.sg (Y.S.); naoki_yamamoto@nuhs.edu.sg (N.Y.)

**Keywords:** marine organism, halisulfate 3, suvanine, hepatitis C virus, NS3 helicase, dengue virus

## Abstract

Hepatitis C virus (HCV) is an important etiological agent that is responsible for the development of chronic hepatitis, liver cirrhosis, and hepatocellular carcinoma. HCV nonstructural protein 3 (NS3) helicase is a possible target for novel drug development due to its essential role in viral replication. In this study, we identified halisulfate 3 (hal3) and suvanine as novel NS3 helicase inhibitors, with IC_50_ values of 4 and 3 µM, respectively, from a marine sponge by screening extracts of marine organisms. Both hal3 and suvanine inhibited the ATPase, RNA binding, and serine protease activities of NS3 helicase with IC_50_ values of 8, 8, and 14 µM, and 7, 3, and 34 µM, respectively. However, the dengue virus (DENV) NS3 helicase, which shares a catalytic core (consisting mainly of ATPase and RNA binding sites) with HCV NS3 helicase, was not inhibited by hal3 and suvanine, even at concentrations of 100 µM. Therefore, we conclude that hal3 and suvanine specifically inhibit HCV NS3 helicase via an interaction with an allosteric site in NS3 rather than binding to the catalytic core. This led to the inhibition of all NS3 activities, presumably by inducing conformational changes.

## 1. Introduction

An estimated 150 million people worldwide are chronically infected with the hepatitis C virus (HCV), a major etiological agent responsible for the development of chronic hepatitis, liver cirrhosis, and hepatocellular carcinoma (World Health Organization, 2013). The current standard therapy is based mainly on a triple combination of pegylated interferon-alfa, ribavirin, and a recently approved NS3 serine protease inhibitor (such as telaprevir), which increases the viral clearance rate to >70% [1,2]. However, because of severe side effects, the emergence of drug-resistant HCV mutations, and drug-drug interactions [3,4], the development of novel direct-acting antivirals that target the viral or host proteins involved in HCV replication are needed urgently. HCV nonstructural protein 3 (NS3) helicase has been considered as a novel antiviral target owing to its essential role in viral replication [5,6].

HCV is a member of the *Flaviviridae* family of positive-stranded RNA viruses. The viral genome contains a single open reading frame encoding a polyprotein that is processed by virus-encoded and host cellular proteases into structural and nonstructural proteins. The structural proteins (core protein [C], and the envelope glycoproteins E1 and E2) build up the virus particle, whereas the nonstructural proteins p7 and NS2 support particle assembly without being incorporated into the viral particles [7,8]. The remaining nonstructural proteins (NS3, NS4A, NS4B, NS5A, and NS5B) form a complex with viral RNA to support viral replication [9]. NS3 is a multifunctional enzyme with serine protease and NTPase/helicase domains at the *N*- and *C*-termini, respectively [10]. The NS3 helicase can unwind double-stranded RNA (dsRNA), double-stranded DNA, and RNA/DNA heteroduplexes in a 3′–5′ direction by using a nucleoside triphosphate as the energy source [11,12,13,14]. Although the exact role of NS3 helicase in the viral life cycle remains unclear, a fully functional NS3 helicase is required for replication of the HCV replicon [5] and for HCV replication in chimpanzees [15], suggesting that NS3 helicase inhibitors could be potential therapeutic agents. However, no HCV NS3 helicase inhibitors have yet been entered into clinical trials, at least in part due to similarities between NS3 and cellular RNA helicases [8].

HCV NS3 helicase is part of the family of viral DExH proteins; the NS3/NPH-II family that encompasses helicases from positive-stranded RNA viruses [16,17,18]. These closely related helicases share a catalytic core that consists mainly of NTPase and nucleic acid binding sites, as well as many other structural and functional features. Indeed, dengue virus (DENV) NS3 helicase, another viral DExH protein, and HCV NS3 helicase share highly conserved amino acid sequences, and consequently have similar conformational structures [19]. Thus, if a compound inhibits HCV NS3 helicase, it may also inhibit DENV NS3 helicase [20,21,22]. Assessing the inhibitory specificity can provide useful information to understand whether inhibitors target the NTPase, nucleic acid binding, or other allosteric sites of NS3 helicase.

HCV NS3 helicase inhibitors function by inhibiting NTP binding, nucleic acid binding, NTP hydrolysis or NDP release, the coupling of NTP hydrolysis to the translocation and unwinding of nucleic acids, or unwinding by sterically blocking helicase translocation [6]. In addition, owing to an interdependent linkage between NS3 helicase and serine protease activities [23,24,25], the inhibition of NS3 serine protease may also lead to the inhibition of NS3 helicase. Compounds that intercalate into the strands of double-stranded nucleic acids could also inhibit NS3 helicase [26].

Naturally occurring products are an important source of structurally diverse and biologically active secondary metabolites. The diversity of organisms in the marine environment has provided new drugs in almost all therapeutic areas [27,28,29]. To date, seven therapeutic agents derived from the marine environment are used as anticancer, antiviral, pain control, and hypertriglyceridemia agents [27]. The chemical structure has been isolated for two of these compounds, whereas the remaining five are synthetic agents based on marine products. An additional 13 agents are in phase 1, 2, or 3 clinical trials. Therefore, natural marine products include a number of highly significant lead compounds that are driving new drug development.

In this study, we screened extracts from marine organisms for NS3 helicase inhibitors using a fluorescence helicase assay based on photoinduced electron transfer (PET), as described in our previous study [30]. During purification, halisulfate 3 (hal3) and suvanine, which were isolated from marine sponge extracts, were identified as novel NS3 helicase inhibitors with IC_50_ values in the low micromolar range. The inhibitory effects of hal3 and suvanine against the other helicase-related activities of NS3 (ATPase, RNA binding, and serine protease activities) were also assessed. Finally, the inhibitory activities of hal3 and suvanine against DENV NS3 helicase were determined to characterize the binding sites of hal3 and suvanine.

## 2. Results and Discussion

**Figure 1:**
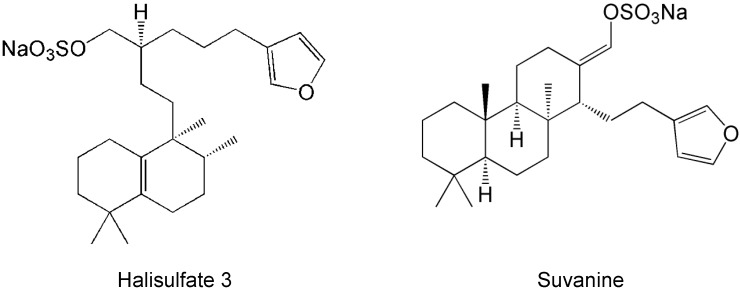
Structures of halisulfate 3 (hal3) and suvanine.

**Figure 2:**
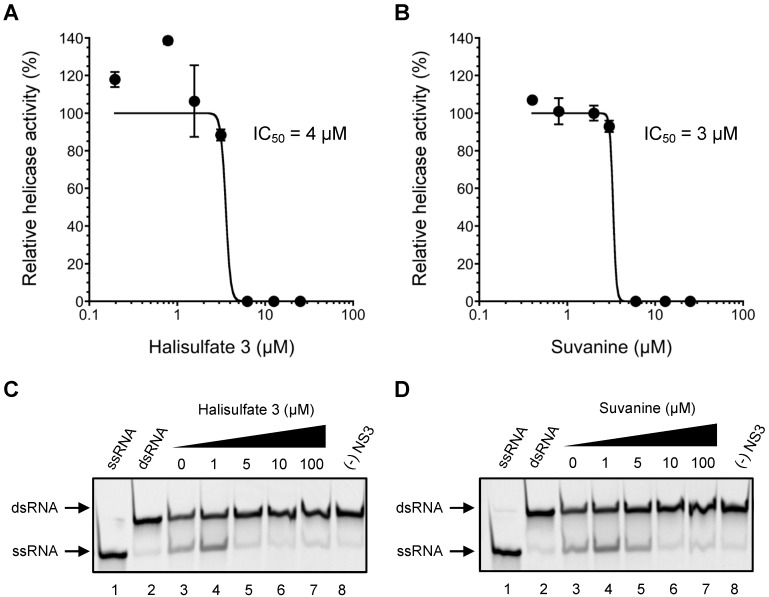
Inhibition of NS3 helicase-catalyzed RNA unwinding activity by hal3 and suvanine. (**A**,**B**) Inhibition curves of hal3 and suvanine generated using a fluorescence helicase assay. The NS3 helicase activities of samples containing inhibitor were calculated relative to control samples containing DMSO vehicle rather than inhibitor. The data are presented as mean ± standard deviation of three replicates; (**C**,**D**) Gel images representing the inhibitory effects of hal3 and suvanine in a gel-based helicase assay. Fluorescence-labeled ssRNA and dsRNA were applied to lanes *1* and *2*, respectively. The dsRNA was incubated with NS3 in the presence of increasing concentrations of inhibitor (lanes *3–7*, 0–100 µM). Lane *8* shows the control reaction in the absence of NS3.

To obtain novel NS3 helicase inhibitors, extracts from marine organisms were screened using a fluorescence helicase assay based on PET. Forty-three extracts prepared from marine organisms were screened, and 11 were identified that inhibited the helicase activity >50% (samples 4, 10, 13, 14, 17, 19, 21, 22, 25, 26, and 37) (Table 1), suggesting that these extracts contained NS3 helicase inhibitors. Of these extracts, sample 10 exhibited the strongest inhibition of NS3 helicase, and abolished its activity completely. Therefore, this extract was purified to isolate and concentrate the inhibitory components. After several purification steps, the inhibitory components were identified as hal3 and suvanine (Figure 1) by comparing their NMR spectra with those reported previously [31,32] for each compound (Supplementary Figures S1–S4). Hal3 and suvanine inhibited NS3 helicase activity in a dose-dependent manner, with IC_50_ values of 4 and 3 µM, respectively (Figure 2A,B).

**Table 1:** Inhibitory effects of extracts from marine organisms on hepatitis C virus (HCV) nonstructural protein 3(NS3) helicase activity.

No.	NS3 Helicase Activity (% of Control) *	Marine Organism	Species
1	92	Sponge	*Unidentified*
2	74	Soft coral	*Briareum*
3	57	Tunicate	*Unidentified*
4	36	Sponge	*Liosina*
5	54	Sponge	*Unidentified*
6	71	Sponge	*Xestospongia*
7	77	Sponge	*Epipolasis*
8	110	Sponge	*Unidentified*
9	86	Sponge	*Strongylophora*
**10**	**0**	**Sponge**	***Unidentified***
11	83	Sponge	*Stylotella aurantium*
12	78	Sponge	*Epipolasis*
13	25	Sponge	*Unidentified*
14	43	Sponge	*Hippospongia*
15	75	Sponge	*Unidentified*
16	85	Sponge	*Unidentified*
17	49	Sponge	*Xestospongia testudinaria*
18	69	Sponge	*Unidentified*
19	40	Sponge	*Theonella*
20	64	Sponge	*Unidentified*
21	44	Sponge	*Unidentified*
22	46	Sponge	*Petrosia*
23	72	Tunicate	*Unidentified*
24	61	Sponge	*Unidentified*
25	50	Tunicate	*Didemnum molle*
26	33	Sponge	*Unidentified*
27	67	Sponge	*Unidentified*
28	87	Soft coral	*Unidentified*
29	62	Sponge	*Unidentified*
30	60	Sponge	*Unidentified*
31	85	Sponge	*Cinachyra*
32	70	Sponge	*Liosina*
33	68	Sponge	*Unidentified*
34	58	Sponge	*Unidentified*
35	72	Sponge	*Stylotella*
36	57	Sponge	*Unidentified*
37	39	Sponge	*Unidentified*
38	72	Tunicate	*Didemnum*
39	62	Sponge	*Unidentified*
40	71	Jellyfish	*Unidentified*
41	74	Sponge	*Unidentified*
42	52	Tunicate	*Unidentified*
43	67	Annelid	*Unidentified*

* NS3 helicase activity in the presence of extract is expressed as a percentage of control in the absence of extract (100%); The sample with the strongest inhibition against NS3 helicase is in bold, underlined font; samples with relatively strong inhibition against NS3 helicase (<50%) are underlined.

The inhibitory effects of hal3 and suvanine were confirmed using a gel-based helicase assay. The helicase activity was calculated as the ratio of the signal intensity derived from single-stranded (ssRNA) in the sample containing the inhibitor to the control sample (lacking the inhibitor but containing DMSO vehicle). Similar to the results of the fluorescence helicase assay, hal3 and suvanine inhibited helicase-catalyzed RNA unwinding in a dose-dependent manner (Figure 2C,D). Therefore, these data clearly indicate that hal3 and suvanine exert inhibitory effects. Hal3 and suvanine were identified in 1988 [33] and 1985 [34], respectively. They have similar distinguishing structural features of a sulfated side chain and a furan moiety at the terminus of the molecule (Figure 1). Although some bioactivities for hal3 and suvanine have been reported, this report is the first that identifies these compounds as helicase inhibitors. In addition, bioactive effects of hal3 alone have not been reported. A mixture of halisulfates 2–5 (hal3 and its analogues) showed antimicrobial activity against *S. aureus*, *C. albicans*, and *B. subtilis*. Moreover, a mixture of halisulfates 2–4 inhibited PMA-induced inflammation in a mouse ear edema assay and inhibited phospholipase A_2_ [31]. Suvanine is a serine protease inhibitor [35] and an antagonist of the mammalian bile acid sensor farnesoid-X-receptor [36]. In addition, suvanine interferes with heat shock protein 60, a chaperone involved in the inflammatory response, giving evidence for its anti-inflammatory properties [37].

**Figure 3:**
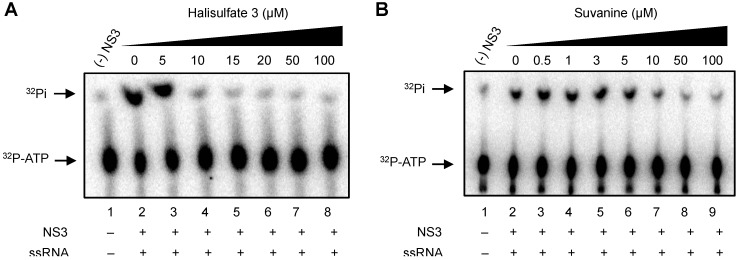
Effects of hal3 and suvanine on NS3 ATPase activity demonstrated by autoradiography of an ATPase assay using [γ-^32^P] ATP. Lane *1* contains the control reaction without NS3. Lanes *2–8* (**A**) and *2–9* (**B**) show the ATP hydrolysis reaction with poly(U) RNA at increasing concentrations (0–100 µM) of hal3 and suvanine, respectively.

As the unwinding ability of NS3 helicase is dependent on ATP hydrolysis, the amount of inorganic phosphate (Pi) released from radioisotope-labeled ATP was measured to determine the effects of hal3 and suvanine on the ATPase activity of NS3 (Figure 3). The released Pi was separated by thin-layer chromatography and visualized using autoradiography. The density of the upper spots corresponding to Pi, which represents ATPase activity, decreased dose-dependently for both hal3 and suvanine. The ATPase activity was calculated as the ratio of the signal intensity derived from the released Pi in the sample containing inhibitor to that in the control sample (lacking the inhibitor but containing DMSO vehicle). The IC_50_ values of hal3 and suvanine were calculated to be 8 and 7 µM, respectively. As this concentration range is similar to that in which RNA unwinding was inhibited (Figure 2), it is likely that hal3 and suvanine inhibit NS3 helicase via the inhibition of ATPase activity.

**Figure 4:**
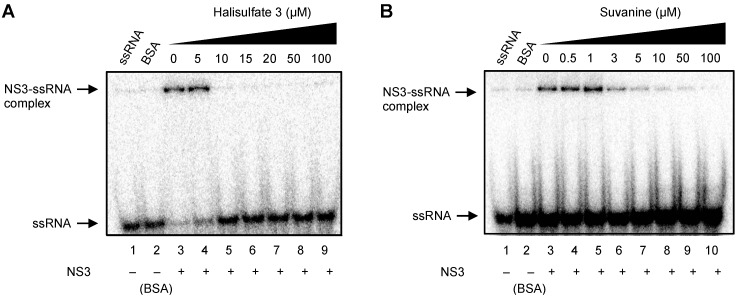
Effects of hal3 and suvanine on NS3 RNA binding activity, assessed by autoradiography of a gel mobility shift assay using ^32^P-labeled ssRNA. Lanes *1* and *2* contain control reactions consisting of heat-denatured ssRNA and 300 nM BSA instead of NS3, respectively. Lanes *3−9* (**A**) and *3−10* (**B**) show the RNA binding reaction with increasing concentrations (0−100 µM) of hal3 and suvanine, respectively.

As RNA binding is required for NS3 helicase activity, the effects of hal3 and suvanine on NS3 RNA binding activity were examined by gel mobility shift assay (Figure 4). As a control, the non-specific binding of ssRNA to bovine serum albumin (BSA) was assessed (lane 2). The density of the upper bands corresponding to the NS3-ssRNA complex, which represents NS3 RNA binding activity, decreased dose-dependently in the presence of both hal3 and suvanine. RNA binding activity was calculated as the ratio of the signal intensity derived from the NS3-ssRNA complex in the sample containing the inhibitor to that in the control sample (lacking the inhibitor but containing DMSO vehicle). The IC_50_ values of hal3 and suvanine were calculated to be 8 and 3 µM, respectively. The data presented in Figure 2 and Figure 4 reveal that the NS3 helicase and RNA binding activities decrease at similar inhibitor concentration ranges for hal3 and suvanine, suggesting that the inhibition of NS3 helicase by these compounds is associated with RNA binding activity.

**Figure 5:**
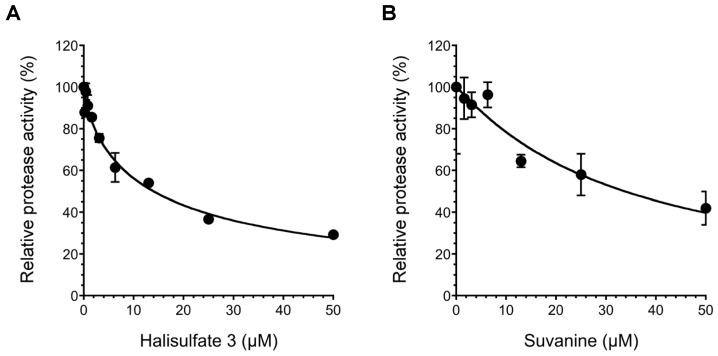
Effects of hal3 (**A**) and suvanine (**B**) on NS3 serine protease activity. The NS3 serine protease activity of samples containing inhibitor was calculated relative to control samples containing DMSO vehicle rather than inhibitor. The data are presented as means ± standard deviation of three replicates.

It was reported that the helicase activity of NS3 is interdependently linked to its serine protease activity [23,24,25]. Therefore, we examined the effects of hal3 and suvanine on NS3 serine protease activity using a fluorescence serine protease assay (Figure 5). Serine protease activity decreased in a dose-dependent manner in the presence of hal3 and suvanine, with IC_50_ values of 14 and 34 µM, respectively. Although the inhibition of the serine protease activity seems to be rather modest compared with that of the ATPase and RNA binding activities (Figure 3 and Figure 4), the inhibition of NS3 helicase by hal3 and suvanine is likely to be also related to serine protease activity.

**Figure 6:**
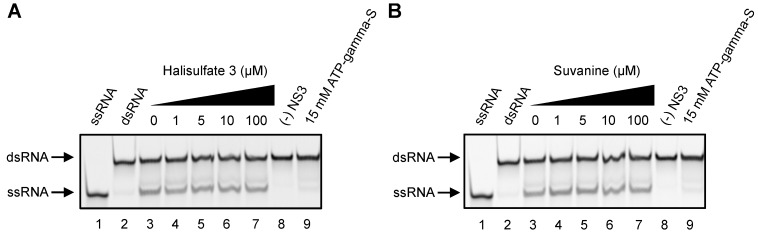
Effects of hal3 (**A**) and suvanine (**B**) on DENV NS3 helicase activity, assessed using a gel-based helicase assay. Fluorescence-labeled ssRNA and dsRNA were applied to lanes *1* and *2*, respectively. The dsRNA was incubated with NS3 in the presence of increasing concentrations of inhibitor (lanes *3–7*, 0–100 µM). Lanes *8* and *9* contain the control reaction mixtures in the absence of NS3, and in the presence of 15 mM ATP-gamma-S as an inhibition control, respectively.

The catalytic cores of DENV and HCV NS3 helicases, which consist predominantly of ATPase and RNA binding sites, share almost identical folds and extensive structural similarity [38]. Because the substrate specificity of DENV and HCV NS3 helicases is similar [39], the dsRNA substrate and capture strand of the gel-based HCV NS3 helicase assay were also used for the gel-based DENV NS3 helicase assay (Figure 6), and helicase activity was calculated as described above. Hal3 and suvanine did not abolish DENV NS3 helicase activity, even in the presence of 100 µM of each inhibitor. This finding suggests that the inhibitory effects of hal3 and suvanine are specific to HCV NS3 helicase, and that these inhibitors bind less efficiently to any site in DENV NS3 helicase, including the catalytic core.

This study demonstrated that hal3 and suvanine inhibit the ATPase, RNA binding, and serine protease activities of NS3 (Figure 3, Figure 4 and Figure 5). Taken together with observations that hal3 and suvanine did not inhibit DENV NS3 helicase (Figure 6), it is likely that these inhibitors do not bind to the catalytic core that contains the ATPase activity and RNA binding sites. Therefore, we conclude that hal3 and suvanine inhibit HCV NS3 helicase via interactions with allosteric sites of NS3. This likely induces conformational changes in NS3, inhibiting or abolishing its activities. Compounds with inhibitory activities against both helicase and serine protease activities have been reported previously [40]; however, there are only a small number of studies, and detailed inhibitory mechanisms are yet to be elucidated. The possible allosteric sites to which hal3 and suvanine bind could be an interface that forms between the helicase and protease domains of NS3. Indeed, a novel small-molecule binding site at the interface between these two domains was reported recently [41]. Furthermore, the inhibitory specificity of hal3 and suvanine against HCV NS3 helicase might be explained by structural differences between HCV and DENV NS3 helicases. A specific beta-strand tethers the *C* terminus of the helicase domain to the protease domain of HCV NS3, maintaining it in a compact conformation that differs from the extended conformation of DENV NS3 helicase [42]. As only HCV NS3 helicase forms an interface between the helicase and protease domains, the specificity of hal3 and suvanine for HCV NS3 helicase would be explained by the binding of hal3 and suvanine to the interface of HCV NS3.

## 3. Experimental Section

### 3.1. Preparation of Extracts from Marine Organisms

Specimens of marine organisms were collected by scuba diving in Okinawa, Japan, and Sorong, Indonesia, and kept frozen until use. The specimens were chopped into small pieces, and soaked in acetone for 20 h followed by methanol for 6 h. The acetone and methanol solutions were then combined and concentrated, and residual materials were separated into ethyl acetate and aqueous layers; each layer was then dried to obtain residues.

### 3.2. Screening for HCV NS3 Helicase Inhibitors

The PET-based fluorescence helicase assay was performed as described previously [30]. The dsRNA substrate was prepared by annealing the 5′ BODIPY FL-labeled fluorescence strand (5′-CUAUUACCUCCACCCUCAUAACCUUUUUUUUUUUUUU-3′) to the quencher strand (5′-GGUUAUGAGGGUGGAGGUAAUAG-3′) at a 1:2 molar ratio. The dsRNA substrate contains the 3′-overhang that is necessary for the NS3 helicase to bind RNA prior to duplex unwinding. The capture strand (5′-CTATTACCTCCACCCTCATAACC-3′), which is complementary to the quencher strand, prevents the unwound duplexes from reannealing. None of the capture, quencher, or fluorescence strands are self-complementary. The fluorescence strand was purchased from J-Bio 21 Corporation (Tokyo, Japan), and was labeled with BODIPY FL at the 5′-end via an aminohexylphosphate linker with a six-carbon spacer. The quencher and capture strands were purchased from Japan Bio Services (Saitama, Japan). The reaction mixture contained 25 mM MOPS-NaOH (pH 6.5), 3 mM MgCl_2_, 2 mM dithiothreitol, 4 U RNasin (Promega, Madison, WI, USA), 50 nM dsRNA substrate, 100 nM capture strand, 5 mM ATP, a marine organism extract, and 240 nM NS3 in a total reaction volume of 20 µL. Each marine organism extract diluted in DMSO was added to the reaction mixture at a final concentration of 17.5–32.5 µg/mL. The full-length HCV NS3 protein with serine protease and NTPase/helicase activities was expressed and purified as described previously [43].

The reaction was started by the addition of HCV NS3 helicase, and was performed at 37 °C for 30 min using a LightCycler 1.5 (Roche Diagnostics, Basel, Switzerland). The fluorescence intensity was recorded every 5 s from 0 to 5 min, and then every 30 s from 5 to 30 min. Helicase activity was calculated as the initial reaction velocity relative to control (in the absence of a marine extract, but presence of DMSO vehicle). The IC_50_ was calculated using KaleidaGraph (Synergy Software, Reading, PA, USA) by fitting plots of % activity *vs*. [*I*] using Equation (1) unless otherwise noted [44]:

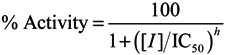
(1)
where *h* is the Hill coefficient, and [*I*] is the inhibitor concentration.

### 3.3. Gel-Based HCV NS3 Helicase Assay

A gel-based helicase assay was performed on HCV NS3 helicases using an Alexa Fluor 488-labeled dsRNA strand and capture strand with the same nucleic acid sequences described in Section 3.2. The dsRNA substrate was prepared by annealing the 5′ Alexa Fluor 488-labeled strand (5′-CUAUUACCUCCACCCUCAUAACCUUUUUUUUUUUUUU-3′) to the complementary strand (5′-GGUUAUGAGGGUGGAGGUAAUAG-3′) at a 1:2 molar ratio. The same capture strand described in Section 3.2 was used. All nucleic acid strands were purchased from Japan Bio Services (Saitama, Japan). The reaction mixture for HCV NS3 helicase contained the same components as described in Section 3.2, with increasing concentrations of hal3 or suvanine in a reaction volume of 20 µL. The reaction was started by the addition of HCV NS3 helicase, and performed at 37 °C for 60 min using a GeneAmp PCR System 2700 (Applied Biosystems, Foster City, CA, USA). The reaction was stopped by the addition of 5 µL of helicase termination buffer containing 10 mM Tris-HCl (pH 7.5), 50 mM EDTA, 30% glycerol, 0.06% bromophenol blue, and 0.12% Orange G. The inhibition of NS3 helicase was analyzed on a 20% native Tris/borate/EDTA (TBE) polyacrylamide gel, and labeled RNAs were visualized using Typhoon 9210 (GE Healthcare, Waukesha, WI, USA). The helicase activity was calculated as the ratio of the signal intensity derived from ssRNA in the sample containing inhibitor to that in the control sample containing DMSO vehicle instead of inhibitor.

### 3.4. ATPase Assay

NS3 ATPase activity was determined directly by monitoring [γ-^32^P] ATP hydrolysis by thin-layer chromatography, as described previously [45,46]. The reaction mixture contained 25 mM MOPS-NaOH (pH 7.0), 1 mM dithiothreitol, 5 mM MgCl_2_, 5 mM CaCl_2_, 1 mM [γ-^32^P] ATP (Muromachi Yakuhin, Tokyo, Japan), 300 nM NS3, 0.1 µg/µL poly (U) ssRNA (Sigma-Aldrich, St. Louis, MO, USA), and increasing concentrations of hal3 or suvanine in a volume of 10 µL. The reaction was conducted at 37 °C for 10 min, and stopped by the addition of 10 mM EDTA. Two microliters of each reaction mixture was then spotted onto a polyethyleneimine cellulose sheet (Merck, Darmstadt, Germany) and developed in 0.75 M LiCl/1 M formic acid solution for 20 min. The cellulose sheet was dried, and the released [γ-^32^P] phosphoric acid was visualized using an Image Reader FLA-9000 and quantified using Multi Gauge software V 3.11 (Fujifilm, Tokyo, Japan). ATPase activity was calculated as the ratio of the signal intensity derived from the released Pi in the sample containing inhibitor to that in the control sample containing DMSO vehicle instead of inhibitor.

### 3.5. RNA Binding Assay

NS3 RNA binding activity was determined by gel mobility shift assay, as described previously [45,46]. The ssRNA (5′-UGAGGUAGUAGGUUGUAUAGU-3′) synthesized by Gene Design (Osaka, Japan) was labeled at the 5′-end with [γ-^32^P] ATP (Muromachi Yakuhin, Tokyo, Japan) using T4 polynucleotide kinase (Toyobo, Osaka, Japan) at 37 °C for 60 min, and purified using the phenol-chloroform extraction method. The reaction mixture contained 30 mM Tris-HCl (pH 7.5), 100 mM NaCl, 2 mM MgCl_2_, 1 mM dithiothreitol, 20 U RNasin Plus (Promega), 300 nM NS3, 0.1 nM ^32^P-labeled ssRNA, and increasing concentrations of inhibitor in a volume of 20 µL. The reaction was performed at room temperature for 15 min. An equal volume of a dye solution containing 0.025% bromophenol blue and 10% glycerol in 0.5× TBE was then added to each reaction mixture, and samples were loaded onto a 6% native-polyacrylamide gel. The labeled RNA bands were visualized using an Image Reader FLA-9000 and quantified using Multi Gauge software V 3.11 (Fujifilm, Tokyo, Japan). RNA binding activity was calculated as the ratio of the signal intensity derived from the NS3-ssRNA complex in the sample containing hal3 or suvanine to that in the control sample containing DMSO vehicle rather than inhibitor.

### 3.6. Serine Protease Assay

A fluorescence NS3 serine protease assay, based on fluorescence resonance energy transfer, was conducted using reagents provided in a SensoLyte™ 520 HCV protease assay kit (AnaSpec, San Jose, CA, USA), as described previously [30]. Briefly, NS3 protein with a two-fold excess of the NS4A cofactor peptide Pep4AK was prepared in 1× assay buffer provided with the kit. HCV NS3/4A protease was mixed with increasing concentrations of inhibitor, and incubated at 37 °C for 15 min. The reaction was started by the addition of 5-FAM/QXL 520 substrate in a 20 µL total reaction volume containing 240 nM HCV NS3/4A protease and increasing concentrations of hal3 or suvanine. Reactions were then incubated at 37 °C for 120 min on a LightCycler 1.5 (Roche Diagnostics, Basel, Switzerland), and the fluorescence intensity was recorded every min for 120 min. NS3 serine protease activity was calculated as the initial reaction velocity in the sample containing inhibitor relative to the control sample containing DMSO vehicle rather than inhibitor.

### 3.7. Gel-Based DENV NS3 Helicase Assay

A gel-based helicase assay was performed using DENV NS3 helicases, and the Alexa Fluor 488-labeled dsRNA strand and capture strand with the same nucleic acid sequences described in the Section 3.3. DENV NS3 helicase requires a single stranded 3′ overhang to unwind dsRNA substrates in the 3′ to 5′ direction [39]; therefore, the substrate specificities of the DENV and HCV NS3 helicases are the same. The reaction mixture contained 50 mM Tris-HCl (pH 7.4), 1 mM DTT, 0.5% Tween 20, 0.25 µg/mL BSA, 2 mM MgCl_2_, 4 U RNasin (Promega), 5 mM ATP, 50 nM dsRNA substrate, 300 nM capture strand, an inhibitor, and 240 nM DENV NS3 in a total volume of 20 µL. DENV NS3 helicase was prepared as described previously [47]. The reaction was started by the addition of DENV NS3 helicase, and was performed at 37 °C for 60 min using a GeneAmp PCR System 2700 (Applied Biosystems, Foster City, CA, USA). The reaction was then stopped by the addition of 5 µL helicase termination buffer that contained 10 mM Tris-HCl (pH 7.5), 50 mM EDTA, 30% glycerol, 0.06% bromophenol blue, and 0.12% Orange G. The inhibition of DENV NS3 helicase was analyzed on a 20% native TBE polyacrylamide gel, and the labeled RNAs were visualized using Typhoon 9210 (GE Healthcare, Waukesha, WI, USA). The helicase activity was calculated as the ratio of the signal intensity from ssRNA in the sample containing inhibitor to that in the control sample containing DMSO vehicle instead of inhibitor.

## 4. Conclusions

This study demonstrated that hal3 and suvanine isolated from a marine sponge inhibited NS3 helicase by suppressing the ATPase, RNA binding, and serine protease activities. Moreover, DENV NS3 helicase, which shares a catalytic core consisting mainly of ATPase and RNA binding activity sites with HCV NS3 helicase, was not inhibited by hal3 or suvanine. Therefore, it can be concluded that hal3 and suvanine inhibit HCV NS3 helicase specifically through interaction with an allosteric site of NS3 rather than the catalytic core, leading to the inhibition of all NS3 activities, presumably by inducing conformational changes. As such, it is possible that hal3 and suvanine are less likely to inhibit other cellular helicases that share a similar catalytic core to HCV NS3 helicase. This provides potentially useful information on advanced drug design strategies to identify novel NS3 helicase inhibitors that are expected to be more specific and less toxic. Experiments to address whether resistant HCV mutants emerge with the use of these compounds are underway in our laboratory.

## Supplementary Files

Supplementary File 1 Supplementary Information (PDF, 137 KB)
