# Supplementary material for: Identification and Biochemical Characterization of Halisulfate 3 and Suvanine as Novel Inhibitors of Hepatitis C Virus NS3 Helicase from a Marine Sponge
Source: Mar Drugs. 2014 Jan 21;12(1):462–76. doi: 10.3390/md12010462 (PMC3917281; doi:10.3390/md12010462)
Supplement: Supplementary File 1 — Supplementary Information (PDF, 137 KB) [file marinedrugs-12-00462-s001.pdf]

Supplementary Information

Figure S1. ^{1}H (**A**) and ^{13}C (**B**) NMR spectra of halisulfate 3 in MeOD.

A

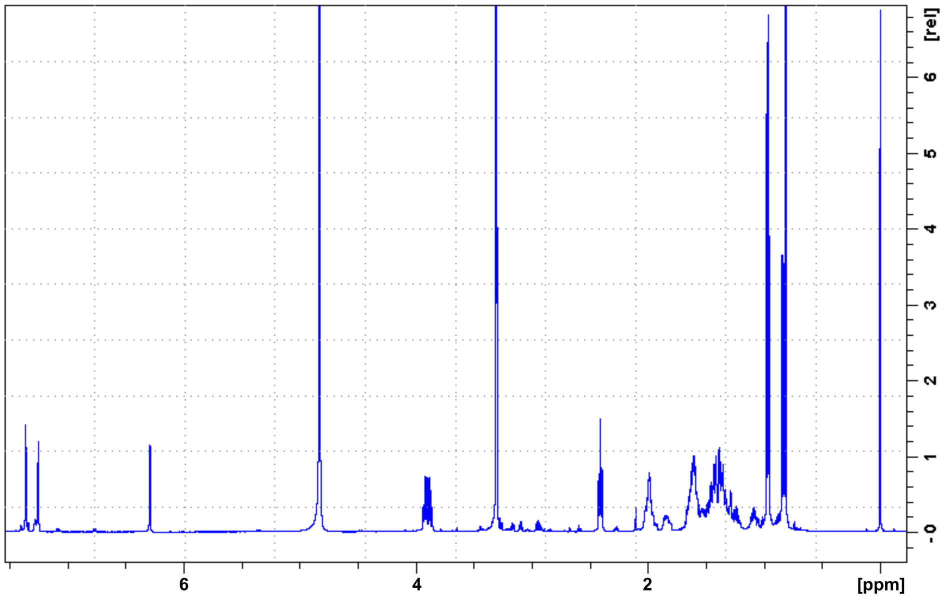

B

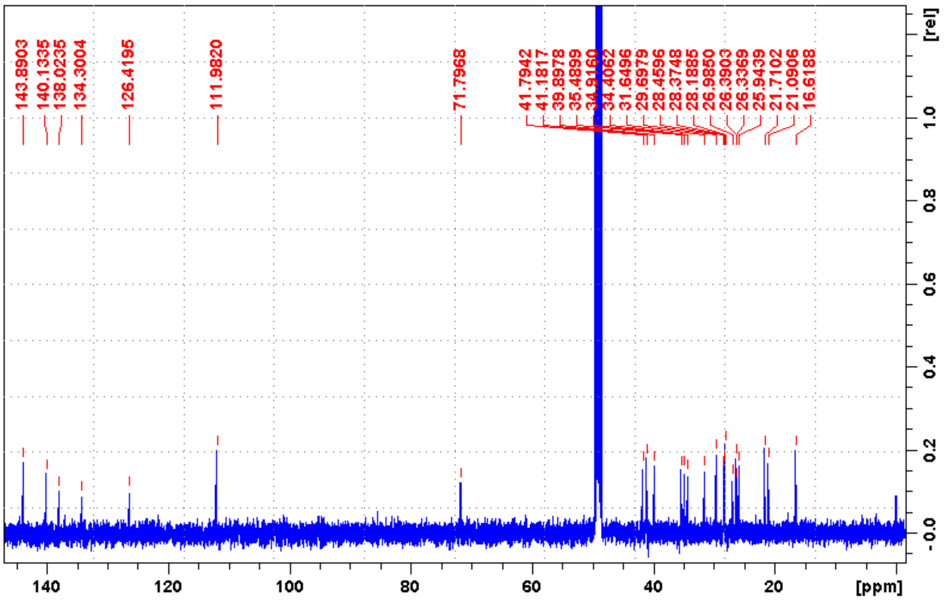

Figure S2. ^{1}H (**A**) and ^{13}C (**B**) NMR spectra of suvanine in $CDCl_3$. The signal at δ 3.5 in the ^{1}H NMR spectra (**A**) is from residual methanol.

A

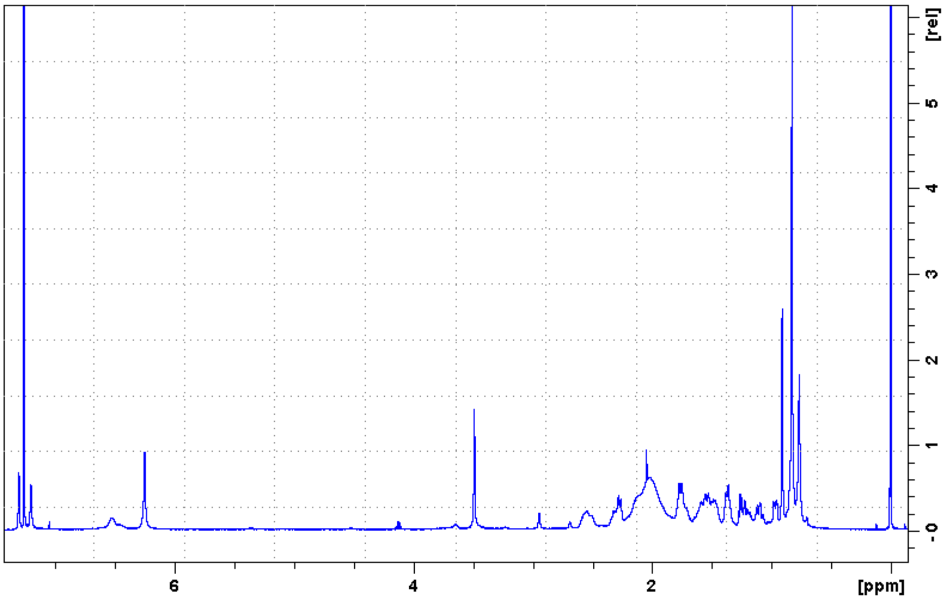

B

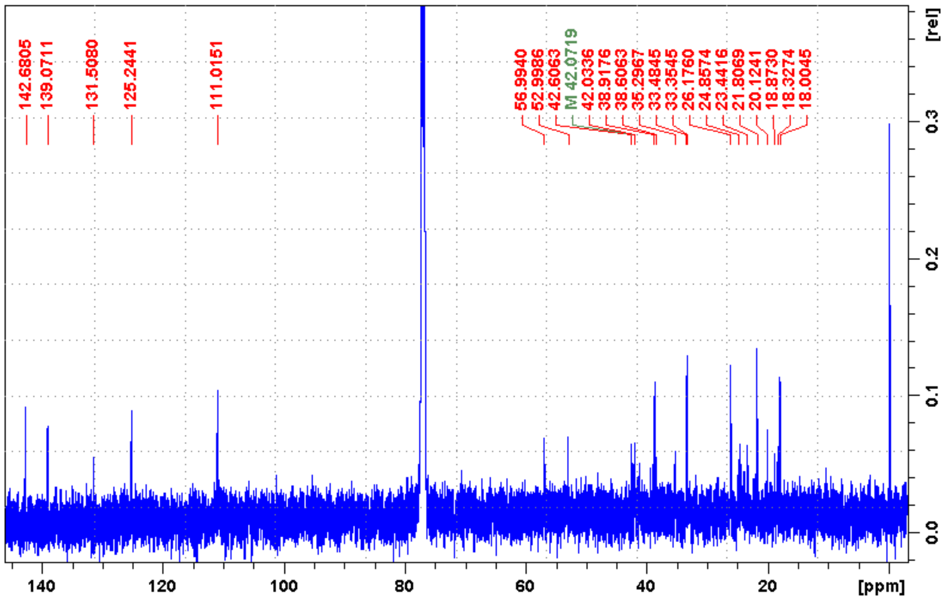

Figure S3. ^{1}H and ^{13}C NMR spectral data for halisulfate 3 in MeOD.

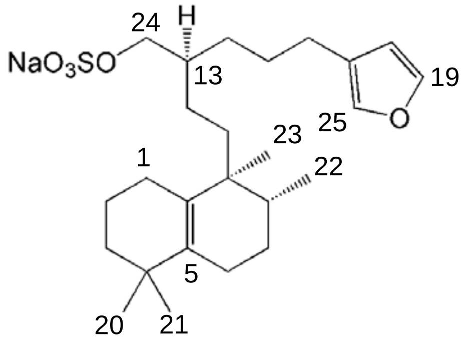

Halisulfate 3

	^{1}H NMR (MeOD)		^{13}C NMR (MeOD)	
C#	Reference [31]	Isolated	Reference [31]	Isolated
1			26.3	26.4 t
2			21.0	21.1 t
3			41.1	41.2 t
4			35.4	35.5 s
5			134.2	134.3 s
6			26.2	26.3 t
7			28.1	28.2 t
8			34.9	34.9 d
9			41.7	41.8 s
10			138.0	138.0 s
11			31.6	31.6 t
12			25.9	25.9 t
13			39.8	39.9 d
14			34.3	34.4 t
15			28.3	28.4 t
16	2.34 t, J = 7 Hz	2.40 t, J = 7.4 Hz	26.9	27.0 t
17			126.3	126.4 s
18	6.24 s	6.29 s	111.9	112.0 d
19	7.32 s	7.36 brs	143.9	143.9 d
20	0.93 s	0.97 s	29.6	29.7 q
21	0.91 s	0.96 s	21.7	21.7 q
22	0.80 d, J = 7.5 Hz	0.83 d, J = 7.0 Hz	28.4	28.5 q
23	0.77 s	0.81 s	16.6	16.6 q
24	3.86 m	3.89 m	71.9	71.8 t
25	7.21 s	7.25 s	140.0	140.1 d

Figure S4. ^{1}H and ^{13}C NMR spectral data for suvanine in $CDCl_3$.

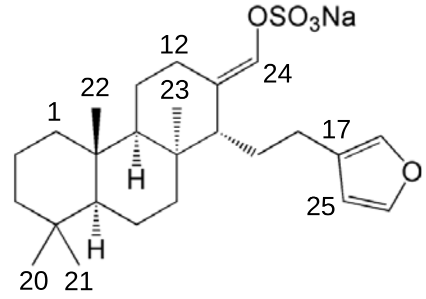

Suvanine

	^{1}H NMR ($CDCl_3$)		^{13}C NMR ($CDCl_3$)	
C#	Reference [32]	Isolated	Reference [32]	Isolated
1			42.0 t	42.0 t
2			18.4 t	18.3 t
3			42.0 t	42.1 t
4			33.5 s	33.4 s
5			53.0 d	53.0 d
6			18.9 t	18.9 t
7			35.4 t	35.3 t
8			39.9 s	38.9 s
9	0.85 m	0.84 m	57.0 d	57.0 d
10			38.6 s	38.6 s
11			20.2 t	20.1 t
12			24.9 t	24.9 t
13			125.2 s	125.2 s
14	2.29 d	2.27 d	42.6 d	42.6 d
15			26.2 t	*
16	2.53 m	2.54 m	23.5 t	23.4 t
	2.32 ddd	2.32 m		
17			126.7 s	*
18	6.32 s	6.24 brs	111.0 d	111.0 d
19	7.23 s	7.20 s	142.6 d	142.7 d
20	0.83 s	0.82 s	33.4 q	33.5 q
21	0.83 s	0.82 s	21.8 q	21.8 q
22	0.92 s	0.91 s	18.1 q	18.0 q
23	0.77 s	0.76 s	26.2 q	26.2 q
24	6.29 s	6.24 brs	131.6 d	131.5 d
25	7.34 s	7.30 s	139.0 d	139.1 d

* Not observed (probably due to short of scans)

© 2014 by the authors; licensee MDPI, Basel, Switzerland. This article is an open access article distributed under the terms and conditions of the Creative Commons Attribution license (http://creativecommons.org/licenses/by/3.0/).
